# Partners in crime: MicroRNA408 and peptide miPEP408 negatively regulate arsenic and low sulfur tolerance

**DOI:** 10.1093/plphys/kiad144

**Published:** 2023-03-04

**Authors:** Yee-Shan Ku

**Affiliations:** Assistant Features Editor, Plant Physiology, American Society of Plant Biologists, USA; School of Life Sciences and Centre for Soybean Research of the State Key Laboratory of Agrobiotechnology, The Chinese University of Hong Kong, Hong Kong SAR, China

MicroRNAs (miRNAs) are RNA molecules consisting of approximately 21 nt and are known for their roles in target transcript cleavage or translational regulation (Axtell and Meyers 2018; Yadav et al. 2021). Primary miRNAs (pri-miRNAs) encode both miRNAs and short peptides that promote the accumulation of the corresponding miRNAs (Yadav et al. 2021), which enhances their regulatory activities.

The regulatory roles of miRNA have been widely discussed. For example, plant miRNAs act as regulators of plant–environment interactions (Song et al. 2019). Arsenic is a common toxic metal pollutant in soil. Accumulation of arsenic in crops not only reduces yield but also introduces arsenic into the food chain (Zhao et al. 2010). Plants adapt to arsenic stress through the promotion of tolerance and the alleviation of toxicity. For example, the glutaredoxin/glutathione (GSH) redox system functions to maintain cellular redox homeostasis under arsenic stress (Bali and Sidhu 2021). To reduce accumulation of arsenic, sulfur metabolism promotes the biosynthesis of phytochelatins that form complexes with arsenic (Bali and Sidhu 2021). Improved tolerance to arsenic could promote crop yield while detoxification of arsenic is beneficial to human and animal health.

In this issue, Kumar et al. (2023) reported microRNA408 (miR408) as well as its encoded peptide miPEP408 as regulators of sulfur assimilation and arsenic stress response in Arabidopsis (*Arabidopsis thaliana*) (Kumar et al. 2023). Whereas miR408 has previously been reported as a regulator of abiotic stresses, miPEP408 has not. By analyzing the sequence upstream from the mature miR408, Kumar et al. (2023) identified a putative open reading frame (ORF) predicted to encode a small peptide of 35 aa (miPEP408-35aa). To demonstrate the regulatory function of miPEP408-35aa, the authors synthesized the small peptide and found its application in the growth medium promoted the growth of Arabidopsis seedlings in the absence of stress. miPEP408-35aa also promoted the accumulation of mature miR408 and pri-miR408 by transcriptional regulation. Using *PromiR408::GUS* transgenic Arabidopsis, the authors showed that miPEP408-35aa enhanced GUS activity. However, under low sulfur condition (LS), arsenic [As(III)] treatment, or LS + As(III) treatment, miPEP408-35aa inhibited seedling growth. To further confirm the negative role of miR408 in seedling growth under these stresses, Arabidopsis lines overexpressing miR408 or carrying knocked out miR408 were generated. The overexpression of miR408 inhibited seedling growth while knockout of miR408 promoted tolerance to these stresses. In addition to the stress tolerance phenotypes, the authors also measured cellular sulfate and GSH levels. Overexpression of miPEP408 promoted the cellular level of sulfate but reduced the cellular level of GSH. Since sulfate is the oxidized form of sulfur, the promoted sulfate level reflects repressed sulfur reduction, an important step for GSH biosynthesis (Kopriva and Rennenberg 2004).

Previously, miR408 was reported to mediate the cleavage of *Plantacyanin* (*ARPN*) and *Laccase-3* (*LAC3*) transcripts (Abdel-Ghany and Pilon 2008). In this study by Kumar et al. (2023), the authors reported *Glutathione S-Transferase TAU 25* (*GSTU25*) as an additional target of miR408. By analyzing the 5′ RNA Ligase Rapid Amplification of cDNA Ends (RLM-RACE) products, the authors showed miR408 mediates the cleavage of *GSTU25* transcripts. The application of miPEP408-35aa also downregulated the expression of *GSTU25*. Overexpression of miR408 downregulated the expression levels of sulfur reduction pathway genes including *Adenosine 5′-Phosphosulfate Sulfurylases* (*ATPS*) and *Adenosine 5′-Phosphosulfate Reductase* (*APR*) in Arabidopsis. The effects of miR408 on gene expression are consistent with the miR408-mediated promotion of cellular sulfate level and the repression of cellular GSH level. Moreover, when miR408 was overexpressed, the reduced cellular GSH level was in line with the increased sensitivity to LS, As(III), and LS + As(III) stresses.

**Figure 1.**
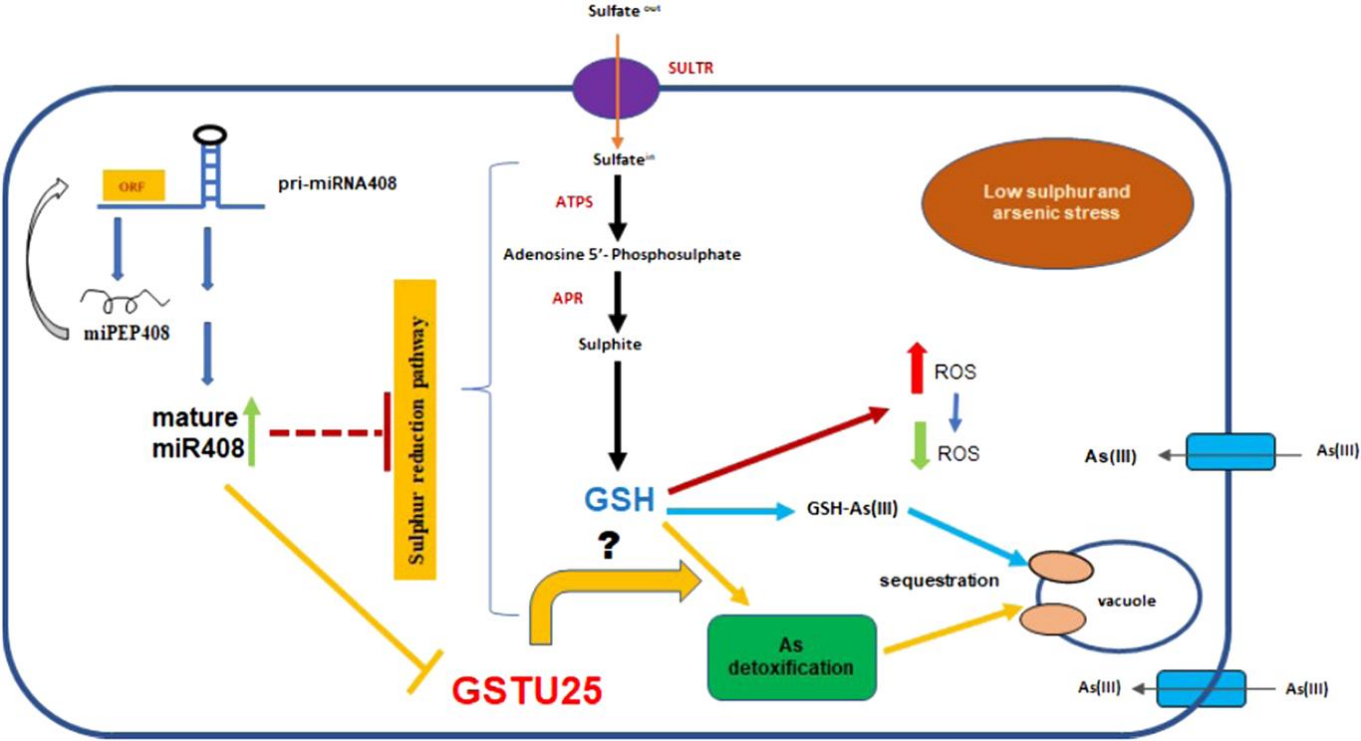
A schematic model showing the functions of miR408 and miPEPE408 in regulating sulfur metabolism under low sulfur and arsenic stress. miPEP408 promotes the level of mature miR408, which meditates the cleavage of *GSTU25* transcripts. miR408 negatively regulates the expression levels of *ATPS* as well as *APR*, and thus the cellular level of GSH. The cellular level of GSH is also reduced under low sulfur condition. The reduced cellular GSH level is negatively associated with As detoxification. ORF, open reading frame; SULTR, sulfur transporter; *ATPS*, *Adenosine 5′-Phosphosulfate Sulfurylases*; *APR*, *Adenosine 5′-Phosphosulfate Reductase*; GSH, glutathione; *GSTU25, Glutathione S-Transferase TAU 25*; ROS, reactive oxygen species. The figure is adapted from Kumar et al. (2023).

In summary, Kumar et al. (2023) showed that miR408 has a profound negative impact on the tolerance to LS, As(III), and LS + As(III) stresses. It is of interest that miR408 has been annotated in more than 30 plants including wheat (*Triticum aestivum*), soybean (*Glycine max*), rice (*Oryza sativa*), barley (*Hordeum vulgare*), and maize (*Zea mays*) (Zhao et al. 2016). The negative role of miR408 in arsenic tolerance shown in this study by Kumar et al. (2023) hints at possible crop improvements by regulating the level of miR408 through the knockout approach. In addition, the authors also demonstrated the possibility to regulate plant growth by applying synthesized peptides. The results supplemented previous reports on miR408 by delineating the role of miPEP408 in promoting the mature miR408 level. The authors further addressed the role of miR408 in regulating LS, As(III), and LS + As(III) stress responses. The finding also enriched the understanding on miR408 by showing that miR408 coordinates LS and As(III) responses. The regulation of LS and As(III) stresses mediated by miPEP408 and miR408 is illustrated in Fig. 1 (Kumar et al. 2023).
